# The mechanism of disaster capitalism and the failure to build community resilience: learning from the 2009 earthquake in L'Aquila, Italy

**DOI:** 10.1111/disa.12431

**Published:** 2020-12-15

**Authors:** Angelo Jonas Imperiale, Frank Vanclay

**Affiliations:** ^1^ Lecturer, Urban and Regional Studies Institute, Faculty of Spatial Sciences University of Groningen The Netherlands; ^2^ Director, Urban and Regional Studies Institute Faculty of Spatial Sciences, University of Groningen The Netherlands

**Keywords:** disaster risk governance, elite capture, organised crime infiltration, rent‐seeking, social dimensions of disasters, social learning, sociology of disasters, transformation towards sustainability

## Abstract

This paper reflects on what materialised during recovery operations following the earthquake in L'Aquila, Italy, on 6 April 2009. Previous critiques have focused on the actions of the Government of Italy and the Department of Civil Protection (Protezione Civile), with little attention paid to the role of local authorities. This analysis sheds light on how the latter used emergency powers, the command‐and‐control approach, and top‐down planning to manage the disaster context, especially in terms of removal of rubble, implementing safety measures, and allocating temporary accommodation. It discusses how these arrangements constituted the mechanism via which ‘disaster capitalism’ took hold at the local and national level, and how it violated human rights, produced environmental and social impacts, hindered local communities from learning, transforming, and building resilience, and facilitated disaster capitalism and corruption. To make the disaster risk reduction and resilience paradigm more effective, a shift from centralised civil protection to decentralised, inclusive community empowerment systems is needed.

## Introduction

Since the 1980s, various international declarations have promulgated a disaster risk reduction (DRR) and resilience paradigm as the guiding basis of disaster management and development agencies in all countries (UNDRO, 1982; IDNDR, 1994; UNISDR, 2005; United Nations, 2015). The framework advocates reducing vulnerabilities and risks and building community resilience during all phases of disaster management and development (IFRC, 2004; UNISDR, 2007; Benson and Twigg, 2007; UNISDR and UNDP, 2007; Department for International Development, 2010; UNDP, 2014). Post‐disaster interventions should be an opportunity to enhance resilience and to build back better not only damaged housing and infrastructure, but also local communities (Quarantelli, 1998; Perry and Quarantelli, 2005; Benson and Twigg, 2007; World Bank, 2009; Hallegatte et al., 2017 ; Hallegatte, Rentschler, and Walsh, 2018; Imperiale and Vanclay, 2020). Understanding how to include community resilience‐building strategies in the design and implementation of post‐disaster interventions should be a key priority of disaster management and development agencies.

For effective DRR and resilience outcomes, there must be transformative, co‐produced knowledge of the multiple dimensions of disaster risks and impacts (Tierney and Oliver‐Smith, 2012; Imperiale and Vanclay, 2019a). To generate community resilience and enhance inclusive social learning and socially sustainable transformation, such knowledge should accompany planned interventions from before conception (Imperiale and Vanclay, 2016b). There must be transparency and accountability, genuine community engagement and empowerment, and effective coordination of myriad stakeholders, with the roles, needs, and priorities of local communities fully recognised and respected (Department for International Development, 2010). Post‐disaster interventions must avoid negative environmental and social impacts, as well as damage to the human rights of affected communities. The individual and collective capacities, emotions (such as empathy), attitudes (such as social responsibility), actions, and behaviours (such as mutual aid), which are conducive to social learning and transformation towards sustainability, must be harnessed and strengthened (IDNDR, 1994; UNISDR, 2005; Benson and Twigg, 2007; Jha et al., 2010; United Nations, 2015; Imperiale and Vanclay, 2016a, 2016b).

For more than 40 years, social science literature pertaining to disaster studies has underlined that post‐disaster interventions exacerbate the inequities that characterise affected local communities, worsening social exclusion and failing to enhance social learning and transformation (see, for example, Oliver‐Smith, 1977, 1990; Bates, 1982; Bolin and Bolton, 1983; Cutter et al., 2006; Gunewardena and Schuller, 2008). Too often, post‐disaster interventions by states result in disaster management agencies exacerbating pre‐crisis vulnerabilities, the root causes of disaster, and associated risks and impacts, with the event being seized as an opportunity for disaster capitalism (Escaleras, Anbarci, and Register, 2007; Gunewardena and Schuller, 2008; Klein, 2008; Owen, 2011; Escaleras and Register, 2016; Faas, 2016; Schuller and Maldonado, 2016; Pyles, Svistova, and Ahn, 2017; Lewis, 2018; Lowenstein, 2018).

Naomi Klein (2005) coined the term ‘disaster capitalism’ to explain the deviant behaviour of the unscrupulous individuals and organisations that extract private advantage in emergency situations. This adverse behaviour is condoned and frequently facilitated by institutional arrangements and neoliberal logic (Gunewardena and Schuller, 2008). The term is widely employed and regularly invoked in analyses of disaster interventions (see, for example, Loewenstein, 2015; Yamada, Cabaljao, and Imasa, 2018; Yee, 2018). Schuller and Maldonado (2016, p. 62) depict it as the ‘instrumental use of catastrophe … to promote and empower a range of private, neoliberal capitalist interests’. Little effort has been made, though, to describe and analyse: (i) the institutional mechanism by which states enable the implementation of disaster capitalism at the local level; (ii) the social risks that allow it to emerge at all levels of society; (iii) the worldview that accompanies it; and (iv) its consequences for local communities' capacities to learn, transform, and build community resilience collectively.

This paper analyses recovery after the earthquake in L'Aquila, Italy, on 6 April 2009, and more specifically, how local authorities used emergency powers, the command‐and‐control approach, and top‐down planning to organise interventions in the affected area. These institutional and financial strategies (in other words, the ‘mechanism‘) of the Italian state distorted the normal physical planning, community participation, and risk management procedures of democratic governance. The paper reflects on how this mechanism permitted disaster capitalism to manifest at all levels of society, rather than facilitating inclusive social learning, transformation, and resilience‐building. In contradiction of the international DRR paradigm, top‐down, military‐type processes were introduced following the earthquake, disrupting local governance, exacerbating local vulnerability to disasters and endemic risks (such as corruption, elite capture, inequity, infiltration of organised crime, public debt, rent‐seeking, and social exclusion), and leading to a failure to build local community resilience. The paper assesses how this happened by scrutinising three topics: (i) disaster governance arrangements; (ii) the business of safety and rubble; and (iii) the establishment of a housing fund called the AQ fund.

## Methodology

This paper is part of a larger research project looking at the social dimensions of the L'Aquila earthquake and the subsequent interventions by the state and civil protection authorities during each phase of disaster management. The primary author, Angelo Jonas Imperiale, is an Italian citizen who was born in L'Aquila and has resided in the central region of Abruzzo for most of his life. He was present in the city of L'Aquila on the night of the earthquake and lived in the mountainous Province of L'Aquila for the next seven years. The larger project utilised a wide range of methods, including: action research; auto‐ethnography; ethnography; participant observation; fieldwork discussions; field notes; field interviews; focus groups; public forums; blogging; surveys immediately following the event; analysis of all relevant documentation; a media review of reporting of the earthquake and its aftermath; 37 retrospective in‐depth interviews with key informants; and more than 250 interviews with people in local communities between 2009 and 2018. The paper draws on these methods, especially on 20 in‐depth interviews that specifically addressed the topics discussed within it.

The 20 interviews were conducted in 2013 (5) and 2017 (15) with local people with knowledge of what happened in the region after the earthquake, and who were willing to speak frankly about their experiences. The participants comprised a member of L'Aquila City Council, the mayors of two mountain villages, six local experts (three seismologists who were closely engaged in local DRR strategies, a lawyer representing the families of the victims, an engineer in charge of the vulnerability reports issued before the earthquake, and a local technician in charge of various rural development programmes in the region), and 11 others who emerged as spokespersons for their various communities. These interviews were recorded and transcribed. Informed consent was obtained and other principles of ethical social research were observed (Vanclay, Baines, and Taylor, 2013).

To avoid formulaic responses, it was decided not to interview people who were strongly associated with the leading political parties, key protest movements, or disaster management agencies. In addition, this paper analyses government and civil protection ordinances and decrees issued by the then President of the Council of Ministers, Silvio Berlusconi, and the then Commander‐in‐Chief of the national Department of Civil Protection (DCP) (Protezione Civile), Guido Bertolaso. It focuses on how these rulings provided local authorities with emergency powers and derogation from ordinary laws and anti‐mafia controls. Also evaluated are mayoral ordinances and decrees concerning the implementation of safety measures and demolition activities, as well as initial reconstruction policies and interventions.

## The earthquake in L'Aquila

A magnitude 6.3 earthquake struck the region of Abruzzo in central Italy at 03:32 on 6 April 2009, devastating the city of L'Aquila and more than 80 villages in 57 municipalities. The event claimed the lives of 309 persons, injured some 1,500 others, and rendered 70,000 people homeless in the affected area, which became known as ‘the crater’. A massive recovery operation was initiated, and many elaborate schemes were implemented. Restricted areas (red zone) patrolled by the military were established almost immediately, excluding people from the town centres. By the evening of 6 April, the DCP had decided to evacuate the crater, which was announced on the radio early the following morning. Within a few weeks, the population was split between the tent camps near L'Aquila (35,856 people) and hotels and other accommodation along the coast of Abruzzo and in other cities in the region (30,124 people). While people were locked out of the historical city centres and scattered along the Adriatic coast and in tent camps, the temporary housing scheme (C.A.S.E. project) was implemented, rubble was managed, and many private building firms carried out interventions, including demolition and shoring‐up solutions, without gaining the consent of homeowners or inhabitants, and thus violating the human right to property and to participate in the reconstruction of homes, villages, and landscapes (Imperiale and Vanclay, 2019b).

The recovery process has been severely criticised by numerous analysts (Frisch, 2010; Alexander, 2010, 2013, 2019; Ozerdem and Rufini, 2013; Calandra, 2016; Contreras, Blasche, and Hodgson, 2017; Imperiale and Vanclay, 2019b). Furthermore, a European Parliament inquiry (S⊘ndergaard, 2013) convened to consider misuse of the EUR 493.7 million provided under the European Union Solidarity Fund. Eleven years after the earthquake, L'Aquila is still a crater, the red zone remains in place, and more than 10,000 people continue to live in temporary accommodation (Barabino and Sansa, 2019).

## The State of Emergency, emergency powers, and new governance of the crater

Within hours of the earthquake on 6 April 2009, Prime Minister Silvio Berlusconi declared a State of Emergency, which provided the DCP with emergency powers, specifically of injunction (to issue ordinances on behalf of the government) and of exception (the derogation of ordinary rules and requirements). Shortly after dawn, the DCP established its local headquarters in L'Aquila, within the Guardia di Finanza (Financial Police) building in Coppito, and created a Directorate of Command and Control (DICOMAC) to manage the emergency. Using various legal measures, including Ordinances of the Presidency of the State, Ordinances of the Presidency of the Council of Ministers (OPCM), and Law and Civil Protection Decrees, emergency powers were deployed without any need for review by parliament.

The DCP had access to the civil protection fund, an emergency reserve that can be drawn upon whenever a State of Emergency is declared. There was very little control over its use, and it was topped up automatically (Law n. 225, art. 5, 24 February 1992). In effect, this gave the DCP relatively unrestricted access to funding. For the three years that the State of Emergency remained in force, all disaster‐related actions and many other initiatives implemented under the guise of the disaster, including attempts to initiate reconstruction and stimulate the economy, were conducted in the absence of standard restrictions and controls, in complete disregard of the norms usually applied to public administration, contracts, outsourcing, and public procurement, and with a complete lack of accountability and transparency. The command‐and‐control approach employed by the DCP did not require any engagement of local communities or their elected councils, and only the local mayors and their trusted technicians were consulted, by‐passing the elected local councils. As the participant councillor said:

*The Italian Constitution … substantially became waste paper, in that … the whole structure of government of the L'Aquila territory was completely overridden. … The municipality's board was never convened, the local council was rendered invisible, was irrelevant, it was cancelled. And this also relates to the relationships local councillors had with DICOMAC. When a local councillor or board member would introduce oneself to a DCP official, the DCP official would not acknowledge any role for that person because the territory was managed only by DICOMAC*.

The elections for the L'Aquila provincial government and other councils within the crater, which would have been held on 6 June 2009, were initially postponed to later in the year and then to March 2010. An ordinance of 15 April 2009 (OPCM, n. 3755, art. 7, subpar. 1 and 2) gave the President of L'Aquila Province, Stefania Pezzopane, authority to implement executively urgent measures. What comprised an urgent measure was not well defined, and this power was applied to a wide range of activities, including what the province is responsible for in normal times. For instance, this power was used to push through the construction of a controversial bridge (Alexander, 2010). The Law Decree n. 39 allocated EUR 200 million to ANAS S.p.A., a state‐owned road construction company under the control of the national Ministry of Infrastructure and Transport, and EUR 100 million to the Italian Railway Network (Rete Ferroviaria Italiana; RFI) to implement actions considered to be necessary for the regional reconstruction process that were within the framework previously established by programme agreements signed before the earthquake. Surprisingly, ANAS S.p.A. also obtained emergency powers ‘to restore with maximum urgency the ANAS offices in L'Aquila’ (OPCM, n. 3755, art. 14, 15 April 2009).

In contrast to the elected councillors, the President of the Abruzzo Region, Gianni Chiodi, and the mayors of the affected municipalities were given enlarged powers. Providing that they deferred to the DCP, they were entitled to (OPCM, n. 3753, 6 April 2009):

commandeer those movable and immovable assets needed to provide refuge and recovery to local inhabitants and purchase all assets and materials needed for local people's sustenance and initial provisional interventions (art. 1, subpar. 1);
identify those structures that could provide adequate refuge to earthquake victims (art. 1, subpar. 2);
implement any urgent or necessary activities to reduce situations of danger and to ensure essential assistance to the affected local population (art. 1, subpar. 3);
establish groups of technicians in each municipality to assess the structural vulnerability of public and private buildings that are totally or partially uninhabitable, or that are not restorable and to be demolished (art. 2, subpar. 1); and
gather the building damage assessments and issue ordinances for demolishment (art. 2, subpar. 2).

The authority of Chiodi was further expanded in Law Decree n. 39 of 28 April 2009, under which he was appointed Delegated Commissioner (Commissario Delegato) and put in charge of implementing urgent interventions (art. 4, subpara. 2), especially concerning the identification of landfill sites for rubble disposal (art. 9, subpar. 8) and the reconstruction of public buildings, including schools, churches, and other cultural heritage properties (art. 4, subpar. 1b). He was supported by the Office of Public Works of the Ministry of Infrastructure and Transport (Provveditorato Interregionale alle Opere pubbliche). The local mayors were given responsibility to coordinate and implement the removal, transport, and disposal of rubble, and to identify new disposal sites.

Although DICOMAC was supposed to provide coordination only for the period of an emergency, it continued to operate until February 2010 (10 months after the earthquake), at which time its responsibilities and authority were taken over by Chiodi and the Mayor of L'Aquila, Massimo Cialente, who respectively became Delegated Commissioner and Deputy Delegated Commissioner for Reconstruction (OPCM, n. 3833, art. 1 and 2). An agency (Struttura Tecnica di Missione; STM) was created to provide technical support and institutional coordination to continue the activities introduced by the DCP and to respond to the ongoing needs associated with reconstruction. Gaetano Fontana, the President of the National Association of Building Firms (Associazione Nazionale Costruttori Edili; ANCE), was controversially appointed as STM Coordinator (Abruzzo24Ore.TV, 2009). The STM came into effect in February 2010, and was supported by: an agency for emergency management (Struttura Tecnica per la Gestione dell'Emergenza; STGE); a techno‐scientific committee; an office for external coordination; and the Office of Public Works of the Ministry of Public Infrastructure and Transport.

The STM structure raised many concerns about its cost and lack of transparency (Orsini, 2011). Polemics arose because the STM imposed the design and implementation of reconstruction plans on local municipalities. These plans were mostly developed by various Italian universities directly appointed by the mayors in controversial circumstances. Suspicions of bribes and corruption led to a legal inquiry in 2012, with Fontana resigning in July 2012 (Nardecchia, 2012). From the perspective of local people, the shift from DICOMAC to STM did not lead to any fundamental change. During the period that the STM was in operation, the State of Emergency was still in force, emergency powers and derogation could still be used, and the views of L'Aquila Council were still not considered, as the councillor explained:

*The local council produced urban planning documents that were separate and different to those produced by Fontana's office. Nevertheless, those produced by Fontana, STM, or DICOMAC were adopted by national decrees issued by Prime Minister Berlusconi or the President of the State, but those produced by our council were not even taken into account*.

The State of Emergency lasted (as noted) for three years, an extraordinarily long period (Venice Commission, 1995; Khakee, 2009; Imperiale and Vanclay, 2019b). However, many decisions taken under this regime, such as about demolitions, safety measures, and construction of infrastructure, continued to be implemented for years afterwards, usually under emergency procedures. The actions of the regional and provincial leaders, and the daily operations of the municipal governments, were not publicly disclosed or monitored, and were covered by state secrecy provisions. As the local councillor stated:

*After 2009, the municipality of L'Aquila stopped producing any financial statements. At the end of every year, the state simply covered any deficit. The local council would say to the state something like: ‘this year we had costs of EUR 30 million for wages, maintenance etc.‘. Without needing to provide a formal budget, the Council would produce a summary of the money spent, and ask the money from the state. Up until last year [2016], the government has always written off our shortfall*.

A controversial aspect concerning the declaration of a State of Emergency in Italy is that it also leads to the suspension of procedures relating to control of mafia organisations. Italy has a long history of trying to control the mafia, with the National Anti‐Mafia Investigative Directorate (Direzione Investigativa Antimafia; DNA) being established in 1991. Over time, anti‐mafia controls have become stricter and since 1991, any firm wishing to tender for public works had to have an anti‐mafia certificate (Ferraro, 2012; Europol, 2013). There were stiff penalties for engaging firms associated with the mafia. Since the mafia had a strong presence in the construction industry, the controls were especially, but not exclusively, directed towards the building sector. The controls required that no employee have any affiliation with organised crime and that the names of all employees and their possible links to organised crime organisations be supplied to the authorities.

With the implementation of the State of Emergency, the anti‐mafia conditions were initially suspended. In response to questioning by journalists, a decree (n. 39, art. 16) was issued on 28 April 2009 to reinstate the anti‐mafia provisions. Unlike all other decrees that applied immediately, though, the anti‐mafia provisions only came into force three months later. This was too late, as safety measures, shoring‐up, demolitions, temporary housing solutions, and rubble removal, transport, and disposal were already being implemented. At least five firms with known mafia connections had been engaged (Galullo, 2009; Libera, 2010; Postiglione, 2010). The European Parliament inquiry (S⊘ndergaard, 2013), the *Annual Report* of the DNA (2017), the Parliamentary Commission of Inquiry into the Mafia (Bindi, 2018), and many other legal probes conducted by the L'Aquila Prosecutor's Office confirmed that there was extensive infiltration by organised crime groups, as well as many irregularities and crimes pertaining to public administration, including fraud, corruption, and bribery (Alexander, 2013; Imperiale and Vanclay, 2019b).

## The business of safety and rubble

The earthquake damaged in excess of 34,000 buildings to some extent, ranging from minor to severe; approximately 37,000 other buildings suffered no damage of consequence. In some cases, buildings were reduced to piles of rubble, or were impacted to such an extent that it was considered not to be practicable to repair them. Some arguably posed a safety threat, which ostensibly was the reason for the creation of the red zone. Immediately after the earthquake, decisions had to be made quickly about whether or not each building could be used. Severely‐damaged buildings that posed a safety risk were slated for demolition. The process of demolition or shoring‐up took place over many years, but principally occurred in the initial months after the disaster.

The first demolitions happened within days of the earthquake, in rather unusual circumstances. On the day of the State Funeral, 10 April, it was announced that a legal inquiry would be convened to identify the contribution of building failure to each of the 309 deaths. Almost immediately, demolition teams moved into the red zone to remove any incriminating evidence. The rubble was transferred to Piazza d'Armi, an underutilised military area close to the city centre, where a pit had been created. Here, two gravel crushers were at work destroying the evidence (Libera, 2010). It took several days before the Prosecutor became aware of the removal of rubble and issued an injunction to stop it. The buildings already under legal inquiry were taped off to protect the evidence within them. One might ask rhetorically: why did the military patrolling the red zone allow teams access to demolish buildings and remove rubble? Did an official give them legitimate authority? And if so, who and why?

The extent of damage meant that there was a mountain of rubble that had to be managed, perhaps three million tonnes (Gabrielli et al., 2018). Streets had to be cleared, and damaged buildings needed to be restored. Rubble was a significant issue in many ways. Debris can have environmental and social impacts, and so it needs to be managed carefully to protect public health and the surroundings (USEPA, 1995, 2008). In Italy, however, waste management is highly problematic owing to the infiltration of organised crime, and because there is no disaster rubble management protocol (Gabrielli et al., 2018). Law Decree n. 39 (art. 9) allowed disaster rubble management to be conducted in Abruzzo without regard for the usual procedures, such as an environmental impact assessment, appraisal and monitoring of risk, safety measures, protection of groundwater at waste disposal sites, and public health and safety standards. Law Decree n. 39 (art. 9, subpara.1, 1‐bis) established that all rubble would be considered as normal urban waste, including biohazardous sewage from the portable toilets in the tent camps and debris from collapsed buildings, even though it would have contained high levels of asbestos and other contaminants (Gabrielli et al., 2018). Law Decree n. 39 (art. 9, subpara. 5) also allowed effectively anyone to open new waste disposal sites, in contrast to the normal procedures governing the fitness of an operator as defined by the national registry of environmental professionals, which, among other things, seeks to prevent infiltration by organised crime. It suspended the ability of the region and province to require mitigation of risk, implement monitoring, or shut down dangerous operations. Furthermore, Law Decree n. 39 (art. 6, subpara. 4‐bis) suspended mitigation and monitoring requirements for water basin and river protection for the whole of the Abruzzo region.

One might think that the presence of rubble would be a vivid reminder of the earthquake, and that a first step towards a return to normalcy would be its removal. An alternative perspective, though, is that the rubble is possibly useful for reconstruction. Given the historic nature of some of the houses, the debris was potentially valuable. The rubble that might be regarded as rubbish or hazardous by some can be seen by local people as prized possessions full of sentiment. People's attachment to their houses and the constituent materials is a driver of participatory reconstruction and enacting inclusive social learning and socially sustainable transformations geared towards reducing vulnerabilities and enhancing DRR and community resilience. Elsewhere, resident involvement in the selection and storage of building materials that could be reused has led to community‐building as part of the reconstruction process (Denhart, 2009). A recent application of the Social Impact Assessment Framework for Action (Imperiale and Vanclay, 2016b) showed how participatory waste management strengthens local community resilience (Little, 2017).

In the crater, however, the risk of organised crime penetration of waste management was high, even before the earthquake (Galullo, 2009; Saviano, 2009). The derogations of normal requirements transformed rubble into an avenue for rent‐seeking, infiltration by organised crime, and disaster capitalism, rather than for participatory reconstruction or building back better more sustainable and resilient societies. The demolition firms were paid for the removal of rubble, and they benefitted too from its sale. Demolitions were carried out without adequate care for the private belongings of inhabitants, including materials that could be reused, such as historic stones and planks. For instance, an old woman lamented during one interview that while a firm was demolishing her house without her knowledge, her historic door, which had been handmade by her grandfather, was taken away. According to eyewitness accounts, the haste at which demolitions were conducted and rubble removed was evidenced by the extent to which personal effects were present among the debris.

In May 2009, the Mayor of L'Aquila nominated a site for rubble storage: a quarry owned by a local construction firm, Teges and Palmerini. Cialente agreed to pay the company EUR 10 million (Libera, 2010), but other firms contested this agreement and a legal inquiry was initiated, which ultimately led to it being rescinded. The DCP intervened and restored the deal (Libera, 2010). The contract was surprising because of the amount of money involved, the lack of transparency of the arrangements, the absence of proper procedure in the awarding of the contract, and the firm's alleged links to the mafia (Libera, 2010).

The first actions in relation to buildings in the red zones were technical surveys to evaluate the *agibilitá* (habitability/liveability) of the damaged structures. The criteria for doing so, which were established as a consequence of previous earthquakes, were reiterated in Ordinance n. 3753 of 6 April 2009. Thousands of professionals from all over Italy registered as DCP volunteers, organised themselves into teams, and began to conduct DCP surveys to gauge *agibilitá*. Separate to these appraisals, the local mayors set up technical teams to identify the buildings in need of safety measures. The mayors and their technical managers directly appointed firms to design and implement safety measures. Less than six months after the earthquake, the entire red zone of L'Aquila city was ‘put into safety’, and it was carved up into districts and assigned to various influential building companies. In the words of one key informant:

*In the L'Aquila red zone, in the first six months after the earthquake, shoring‐up solutions were implemented on almost all buildings. … They [technical teams] determined the buildings that needed to be put into safety, but, practically, it was … almost all buildings. … Then, the local municipality assigned a different zone of the city centre to each of its ‘friends’. Yes! The city was literally split up into different zones so that, if one had to make a map of the city, it would have shown the city divided into these different zones, enabling the construction firms to work comfortably [said with a laugh to imply without interference or oversight]*.

The L'Aquila red zone was ‘put into safety’ with impressive speed and in the form of a reward or gift to influential local building firms. The local councillor said:

*When the process of reconstruction was about to start, the Civil Protection decided to make the big local building firms happy because [the beneficiaries of the temporary housing scheme were mostly external building firms]. … It was clearly time to give something to the local entrepreneurs, building firms etc., who obviously were pressuring the local political representatives*.

Safety measures were implemented via mayoral ordinances, and managed by the technical directors of the councils. As the councillor stated:

*[The Director of Public Works] could appoint private companies just with a phone call. … After a while, this system created suspicions so he decided to establish a ‘white list’ of acceptable building firms. Every firm that wanted to be included had to present an anti‐mafia certificate … he established a time schedule for each assignment. This was true, but the problem was that, for example, to Company A he gave an assignment for EUR 80,000, to Company B an assignment for EUR 160,000, and to Company C an assignment for EUR 3 million. He would contact Company C again for another intervention. If you tried to say, ‘Look, Company C already got the money for the last job’, he replied, ‘Yes, but Company D refused and I know that Company C can do a good job, so I appointed them’. It was clear that this was improper conduct [un mercato indecente], however it was a business accepted by everyone. So much so that, during the trial to defend himself, he said: ‘in front of my office door there were queues of local councillors who were there to ask me to put this or that building firm on our short list’*.

Legitimised by mayoral ordinances, the building firms were in control and could design and implement safety measures without community engagement or public oversight, something which enabled excessive interventions. As one key informant confirmed:

*That there was a general misuse of the safety measures applied to public and private buildings in the whole crater is a matter of fact. Shoring‐up solutions and demolitions were disproportionate; some were totally wrong. They destroyed people's houses that were supposed to be put into safety. They broke the interior of the buildings, even their furniture. They stole everything they could from inside the homes. And this was the result of the lack of control over the whole operation. Or the intentional lack of control*.

In response, the interviewer asked: ‘How did the building firms get appointed?‘. The interviewee retorted: ’Do you want a true answer? Friends of friends, this was the way it worked’.

Building firms could gain access to properties while the owners were locked out of their homes and forced to live in emergency conditions hundreds of kilometres away. The red zones of L'Aquila city and the mountain villages around were delivered into the hands of private building firms. Work in the red zones began without any engagement of local inhabitants, without allowing them to access their buildings or to exercise decisions relating to their property and its future. As a local landlord pointed out:

*The big damage to our village was caused by the building firm that carried out shoring‐up solutions. … Our problems began when the building firm arrived … I had to come back from the Adriatic coast to monitor what they were doing, because from the hotel I could not monitor the situation. … It was only pure coincidence that one day I came back to my house and found a worker putting seals across the entrance—the door to my home, you understand? With all my stuff inside! … I asked, ‘Excuse me, who can explain what is going on?’. One person told me: ‘We are implementing shoring‐up solutions on the houses, we are making the buildings safe’. And I told him: ‘Sorry, that is my home, I have many things inside and you are now closing off the entrance so that I cannot get in anymore’. … He asked me how my home was classified. When I replied ‘E’ [uninhabitable], he said to me: ‘“E”; then sorry, but you cannot do anything’. I answered back, ‘“E” does not mean expropriated, you cannot expropriate my home. The house is mine, you must contact me. I left my telephone number on the door’*.

With regard to how building firms implemented shoring‐up solutions, she reported:

*Once, I hid under a crane, they could not see me but I could see them. I could see how they were conducting their operations implementing their shoring‐up solutions. I saw how they put tie rods on the damaged houses of our village. They used to make, not holes, but chasms inside walls, and while one worker was saying ‘take care’, the other one, who was leading the operation, was screaming to him ‘who cares? Come on. Pull’. I could see how they carried out these operations, the violence, the aggression, and the hatred they used to further destroy the houses. Such vandals, vandals! Believe me, it was horrible … They wanted to get themselves more work, beyond what they already had. Thus, day by day, they got themselves more work on private buildings that did not need any safety measures, but they did shoring‐up solutions anyway. … This building firm occupied our village for two long years. They were donkeys amongst us, they behaved like animals, they mocked us, they laughed in our faces. They were the owners of the village. When they finally went away, only then did I feel safe*.

Over time, many scandals emerged in relation to safety measures. There was no monitoring of design or implementation, or of how contracts and subcontracts were assigned. A senior police detective explained:

*During the six months following the disaster, we clearly made a mistake. All our efforts were focused on the inspections we were instructed to do regarding likely criminal organisation infiltration in the new C.A.S.E. buildings. We did not take into account that there were entire city centres that were declared red zones in which building firms and subcontractors were at work undertaking demolition and implementation of safety measures and shoring‐up solutions. … All our efforts and inquiries focused on the C.A.S.E. project, that's why our inquiries about crimes against public administration concerning safety measure implementation only emerged years later*.

A local online newspaper reported that a legal inquiry into the implementation of safety measures, named ‘*Do ut des*‘, had identified an intricate system of corruption involving ad hoc consulting firms to which building companies made payments (Orsini, 2016). Rather than the classic overnight bag stuffed full of money, the bribes of the new millennium were electronic payments to personal firms for alleged consulting advice. It was evident from the probe that unrestricted access to funding and a complete lack of monitoring constituted ideal conditions in which corruption could flourish. The extent to which safety measures were implemented in L'Aquila and the swiftness of these operations were phenomenal. As the councillor reported:

*Corruption also happened in Umbria. If one talks with those building entrepreneurs who were there, they understand that safety measures implementation was a system of corruption. Here [L'Aquila], it was the same. The difference was in the amount of money spent, because in Umbria they spent little money, while here the cost of safety measures was extreme, also because the whole of L'Aquila city centre was put into safety. This was without any oversight. Thus, if this apartment had to be put into safety, and EUR 20,000 of building materials were sufficient, the building firm would put in EUR 40,000 of materials and no one controlled it or asked why*.

Up to August 2012, when the State of Emergency ended (and even afterwards), local municipalities viewed demolition and safety measures as urgent actions that could be carried out without informing homeowners. Far from ‘putting buildings into safety’, the process caused considerable delay to the start of reconstruction, further marginalising local communities from their properties and the right to decide about their future, and exacerbating social risks and vulnerabilities at the local scale.

Two local building firms were convicted of crimes against public administration in January 2018, relating to the work that they undertook during the State of Emergency. The judgement declared that there was no reason for the work to be done in haste, and that the emergency procedures enabled them to engage in irregular subcontracting, false invoicing, and fraud. These local building firms were among the first to be contracted to implement safety measures, which led to much public discussion and eventually to a parliamentary inquiry after it was revealed that, immediately after the earthquake, the Mayor of L'Aquila, Cialente, and other notable figures had been hosted for several months in a resort owned by one of the entrepreneurs (Faz, 2010). As a result of the inquiry, the resort was confiscated by the Finance Police (Redazione, 2015).

## The AQ ethical fund and the allocation of apartments

There were around 3,000 unoccupied apartments in L'Aquila prior to the disaster in 2009. An urban development plan from 1975 estimated that the population of the city would be roughly 160,000 by 2010. This plan, the only one that L'Aquila ever developed, encouraged local building firms to erect speculative housing. There were enough houses before the earthquake for 100,000 people, even though there were only about 70,000 residents, meaning that some 3,000 apartments stood empty. The local branch of the National Association of Building Entrepreneurs (Associazione Nazionale Costruttori Edili; ANCE) indicated to the DCP that these 3,000 empty apartments, which were largely undamaged by the earthquake and more‐or‐less ready for occupation, could be used for temporary housing. A government ordinance of 15 May 2009 (OPCM, n. 3769) granted the authorities the power to expropriate buildings for the purpose of temporary housing. However, this provoked outrage among local building entrepreneurs, who lobbied heavily for new arrangements.

Local businessman Antonio Napoleone was appointed on 18 May 2009 as an advisor on housing and expropriation to DICOMAC, L'Aquila municipality, and the local prefecture. Napoleone negotiated a complex arrangement involving the establishment of a ‘real estate ethical fund for reconstruction’. This AQ Fund, guaranteed by the state, would purchase the vacant apartments and rent them out to people displaced by the earthquake, with the rent subsidised by the state, at least for some years. The apartments would then be sold, potentially to the people who rented them. The initial proposal entailed promised capital of EUR 100 million, 40 per cent from two state‐owned companies (Fintecna and Fimit), and the remaining 60 per cent from a consortium of banks. The arrangement was facilitated by OPCM n .3789 of 9 July 2009 (art. 5), which provided a state contribution of EUR 30,000 per incomplete apartment to enable it to be made ready for use, and an additional EUR 2,000 for furniture. The initial proposal would have involved the purchase of 500 apartments (bought presumably for EUR 200,000 each), made available to displaced people. Difficulties in securing the capital, though, meant that only 392 apartments were reportedly made available, although a subsequent inquiry revealed that only 350 were actually supplied (Gianforte, 2018).

The scheme drew a lot of criticism in the Italian media. The points of contention concerned, inter alia: a lack of transparency and accountability in relation to the whole operation, including financial arrangements; the absence of a community housing assessment; the procedure by which people were chosen to participate; and a perception that the arrangement was capturing public funds to benefit local private firms and individuals. The councillor described the situation as follows:

*This so‐called ethical real estate fund was supposed to have an ethical form. In reality, it was only a scam to utilise the many unsold buildings of some local building entrepreneurs. … The proposal of this real estate fund actually came from local building entrepreneurs, but this was kept hidden for months. I say kept hidden, because you could not hear anyone talking about this fund. You could hear along the corridors of the local administration that ‘they are doing this real estate fund’, but no one really knew at that time what it really meant. When the first beneficiaries of this temporary housing scheme were announced, you could recognise that they were all the people in the city who had or have had power, obviously because having a proper home was more comfortable than sleeping in a hotel or a tent. The L'Aquila municipality—if we mean by it, its local council—did not know anything about the financial operation of this scheme. Only the local mayor knew about it and approved it. Because the AQ Fund benefitted from a public contribution, and thus the monies it spent were public money, the local mayor should have said something. … There wasn't any public ballot [or fair selection criteria] and when the names of the first beneficiaries came out, as I already said, they were all people of the high bourgeois of the city, or that had or have had power in the city*.

## Discussion: the mechanism enacted by states that facilitates disaster capitalism

The sections above analyse how the Government of Italy provided local authorities with emergency powers to manage disaster rubble, make damaged buildings safe, and determine temporary housing procedures. Extrapolating from the findings, the paper reflects below on how the three top‐down, military‐type arrangements adopted by local and national authorities—emergency powers, command and control, and top‐down planning—constituted the mechanism that facilitated rent‐seeking, elite capture, infiltration by organised crime, and corruption, and enabled disaster capitalism to take hold.

### Emergency powers

The emergency powers allowed national and local authorities to appoint directly suppliers to provide the goods and services needed for emergency accommodation, such as food and portable toilets. In addition, they permitted them to appoint directly staff, consultants, and building firms to construct temporary accommodation, restore public buildings, implement safety measures and reconstruction plans, manage disaster rubble, and erect new infrastructure. The use of emergency powers was deemed necessary because of the perceived urgency of the task and the intention to end the crisis quickly. However, it extended the emergency, granting the elite the opportunity to exploit the post‐earthquake situation. Consequently, the reconstruction of the local physical and social fabric was delayed, and the amount of time that people were compelled to live in emergency conditions was lengthened, thus increasing harm in the short, medium, and long term. Local communities were excluded from the reconstruction process, and their right to decide about the future of their property, village, city, and affected landscape was disrespected.

The emergency powers accorded national and local authorities with state secrecy provisions and led to derogations of ordinary law, including public procurement, anti‐organised crime controls, public health, and environmental safeguards regulations. Although no‐bid contracts in previous disaster contexts had been criticised as avenues for disaster capitalism (Button and Oliver‐Smith, 2008; Damiani, 2008; Klein, 2008), they were utilised considerably in the case of L'Aquila, facilitating the interests of influential building firms and entrepreneurs, exacerbating inequalities, and enabling elite capture and disaster capitalism to flourish. Many decisions taken under the emergency powers regime not only undermined transparency, accountability, and effective community resilience‐building strategies, but also continued to be implemented for years after the earthquake, creating second disasters. The secrecy provisions, the lack of disclosure, and the derogations associated with the State of Emergency have served to hide dubious arrangements, disguise fraud and corruption, and further aided the infiltration of organised crime and disaster capitalism.

### Command and control

The employment of emergency powers in L'Aquila was accompanied by a command‐and‐control approach to resources. This led to the suspension of the democratic functioning of local councils, with only the mayors and their trusted technicians and technical directors having a say in post‐disaster interventions. The Presidents of the Abruzzo Region and L'Aquila Province and the local mayors embodied the command‐and‐control approach in the way that they executed their tasks, including the management of rubble, the introduction of safety measures and demolitions, initial reconstruction, and infrastructure project implementation. A rigid command chain was considered to be necessary at the local level to determine how to spend money efficiently. Instead, though, it facilitated limited public oversight and participation, rent‐seeking, elite capture, corruption, and infiltration of organised crime. Rather than enabling inclusive social learning and socially sustainable transformation, such an approach failed to respect international disaster management principles, allowed disaster capitalism to take root, and worsened local social risks, including inequity and social exclusion.

By promulgating disaster myths, creating perverse opportunities, and failing to introduce adequate oversight mechanisms, the command‐and‐control worldview led to a culture of disaster capitalism, with local people's positive emotions, attitudes, and behaviours being subverted. Empathy was turned into fear and suspicion, social responsibility morphed a gold rush and divinisation of the commander‐in‐charge, and mutual aid was transformed into rent‐seeking and elite capture (Imperiale and Vanclay, 2019b, 2020).

### Top‐down planning

The design of recovery operations in L'Aquila adhered to a top‐down approach to physical planning, which was negatively influenced by the economic interests of national and local elites and did not acknowledge the social dimensions of the interventions (Imperiale and Vanclay, 2020). The top‐down planning pertaining to emergency shelter, temporary housing, safety measures, rubble management, restoration of key public buildings, and the construction of infrastructure was accompanied by techno‐scientific assessments. This resulted in over‐engineered actions that created more problems, worsened local vulnerability to endemic risks, violated human rights, produced environmental and social impacts, and increased public debt. Top‐down planning did not take into account the environmental and social impacts of the interventions or the human rights that had to be respected, further marginalising and fragmenting local communities and generating widespread conflict and discontent, while supporting the interests of national and local elites and failing to build resilience. There was nothing in this system to prevent disaster capitalism from prospering; instead there seemed to be very good knowledge of how to enable elites to hijack the interventions and allow disaster capitalism to thrive.

## Conclusion

The response and recovery operations in the wake of the earthquake in L'Aquila in 2009 were carried out under a regime of emergency powers, command and control, and top‐down planning. These arrangements were the institutional, financial, and physical planning strategies that constituted the mechanism adopted by local and national authorities to implement post‐disaster interventions. The authorities asserted that this mechanism was needed to avoid delays and ensure efficient outcomes. Yet, despite expenditure of approximately EUR 22 billion (Finocchiaro, 2017), 11 years after the earthquake, the red zones still exist all across the crater, and more than 10,000 people still live in temporary housing (Barabino and Sansa, 2019).

Reflecting on the L'Aquila case, and drawing on Klein (2008) and other authors (Gunewardena and Schuller, 2008; Loewenstein, 2015; Faas, 2016; Schuller and Maldonado, 2016), this paper defines disaster capitalism as a broad multidimensional concept that relates to the deliberate, perverse actions of self‐interested parties to extract private advantage from disasters, as well as the mechanism enacted by states that facilitate these actions and protect the elites. Disaster capitalism manifests at all levels of society and during all phases of disaster management. The mechanism comprises cultural and institutional dimensions and includes: the deliberate distortion of information; the promulgation of disaster myths, particularly concerning local communities and matters of urgency; the use of emergency powers, command and control, and top‐down planning; police action and militarisation; and the hijacking of post‐disaster interventions. Disaster capitalism emerges from pre‐existing social risks and vulnerabilities, and enables rent‐seeking, elite capture, infiltration of organised crime, and corruption, creating environmental and social impacts and human rights violations and exacerbating local social risks (such as social exclusion and inequity) and vulnerabilities, while undermining the positive feelings, attitudes, and behaviours that enable members of affected communities collectively to learn, transform, and build resilience (Imperiale and Vanclay, 2016a, 2019b).

The main lesson learned from the disaster in L'Aquila is that crucial changes are required in the way in which states typically conceive their institutional and financial strategies, and their approaches to physical planning, risk management, and community participation. Following the paradigm shift from a war approach to consideration of the social dimensions of disasters (Quarantelli, 1998; Perry and Quarantelli, 2005; Oliver‐Smith et al., 2017), there has been a switch from civil defence to civil protection arrangements (Alexander, 2002). As the case of L'Aquila shows, though, this shift was not accompanied by any substantial alteration to institutional arrangements or management and planning models. Even under a civil protection regime, disaster myths keep accompanying disaster management interventions, and emergency powers, command and control, and top‐down planning remain the mechanism used for disaster recovery and reconstruction operations. This permits disaster capitalism to take hold, instead of enhancing inclusive social learning and socially sustainable transformation and building community resilience.

To enable the various United Nations principles and declarations relating to DRR and resilience to be respected and implemented more effectively in practice, the international community must pay more attention to the mechanism by which states conceive, decide, design, and implement disaster management interventions. The DRR and resilience paradigm demands a shift from protecting vulnerable, affected communities to engaging and empowering their capacities to learn and transform, and thus from centralised, civil protection systems to inclusive, decentralised community empowerment systems. The latter should be capable of developing sustainable governance strategies to prevent disaster capitalism and orient investments and interventions towards reducing local vulnerabilities and environmental and social risks and impacts, enabling and empowering social learning and socially sustainable transformations, and building resilience at all levels of society before and after a disaster.
